# Enhancing Bloodstream Infection Management: A Systematic Review of Rapid Diagnostic Tests and Their Integration Into Antimicrobial Stewardship Programs

**DOI:** 10.7759/cureus.92866

**Published:** 2025-09-21

**Authors:** Shivam Singla, FNU Murk, Bhavna Singla, Sanu Lama, Muhammad Usman Fareed, Nabila N Anika

**Affiliations:** 1 Internal Medicine, TidalHealth Peninsula Regional, Salisbury, USA; 2 Internal Medicine, Peoples University of Medical & Health Sciences for Women, Nawabshah, PAK; 3 Internal Medicine, Erie County Medical Center, Buffalo, USA; 4 Research, Prime Healthcare, Ontario, USA; 5 Internal Medicine, Janaki Medical College and Teaching Hospital, Janakpur, NPL; 6 Medicine and Surgery, Nishtar Medical University, Multan, PAK; 7 Medicine and Surgery, Holy Family Red Crescent Medical College, Dhaka, BGD

**Keywords:** antibiotic optimization, antimicrobial stewardship, bloodstream infections, clinical outcomes, infection management, maldi-tof ms, multiplex pcr, phenotypic susceptibility testing, randomized controlled trials, rapid diagnostic tests

## Abstract

This systematic review evaluates the effect of rapid diagnostic tests (RDTs) on clinical outcomes in patients with bloodstream infections (BSIs), with particular emphasis on their integration into antimicrobial stewardship programs (ASPs). A comprehensive literature search across PubMed, Scopus, Embase, and the Cochrane Central Register of Controlled Trials identified randomized controlled trials (RCTs) and post-hoc analyses that compared RDTs, including multiplex polymerase chain reaction (PCR), matrix-assisted laser desorption ionization-time-of-flight mass spectrometry (MALDI-TOF MS), and rapid phenotypic antimicrobial susceptibility testing (AST), with conventional microbiological workflows. Studies were assessed for methodological quality using the Cochrane risk of bias tool. Outcomes of interest included time to effective therapy, antimicrobial optimization, mortality, and hospital length of stay. The included RCTs consistently demonstrated that RDTs, particularly when paired with ASP interventions, accelerated therapeutic decisions and improved antibiotic targeting compared with conventional methods. Importantly, this review is novel in restricting inclusion to RCT evidence and in examining individual diagnostic modalities, offering more granular insights than prior reviews that pooled heterogeneous study types. However, variability in study design, population characteristics, and outcome definitions highlights the need for standardized research approaches and consistent integration with stewardship frameworks. Overall, the findings support the clinical value of RDTs in enhancing BSI management and optimizing antimicrobial therapy, while underscoring that their impact is maximized when embedded within structured ASPs. These results carry important implications for both high-income and resource-limited healthcare settings, reinforcing the role of diagnostic stewardship in translating rapid results into improved patient outcomes.

## Introduction and background

Bloodstream infections (BSIs), including sepsis and septicemia, represent a significant burden on global health systems, contributing to high morbidity, mortality, and healthcare costs. Sepsis, defined as life-threatening organ dysfunction caused by a dysregulated host response to infection, is a leading cause of death worldwide, particularly in intensive care settings [1,2]. Early identification and prompt administration of effective antimicrobial therapy are essential for improving patient outcomes. Delayed or inappropriate empirical antibiotic treatment is consistently associated with increased mortality and poor prognosis in patients with sepsis and BSIs [3].

Traditionally, blood cultures have served as the gold standard for pathogen identification and antimicrobial susceptibility testing (AST). However, conventional culture methods are time-consuming, often requiring 48 to 72 hours for definitive results [4,5]. This diagnostic delay often necessitates broad-spectrum empirical antibiotic use. Such use may contribute to antimicrobial resistance, unnecessary drug exposure, prolonged hospital stays, and increased healthcare costs. The rise of multidrug-resistant organisms further complicates the management of BSIs, making rapid initiation of targeted therapy increasingly crucial. The time lag in conventional blood culture results has therefore prompted the development of rapid diagnostic tests (RDTs).

RDTs include technologies such as multiplex polymerase chain reaction (PCR), matrix-assisted laser desorption ionization-time-of-flight mass spectrometry (MALDI-TOF MS), T2 magnetic resonance (T2MR), and adjunctive point-of-care biomarkers like procalcitonin (PCT) and C-reactive protein (CRP) [6]. While MALDI-TOF MS provides rapid microbial identification, it generally requires a positive culture before analysis. In contrast, PCT and CRP are indirect biomarkers of infection and inflammation rather than true pathogen-identification tools, though they can support decision-making when used alongside direct diagnostic methods. Several studies have evaluated the clinical impact of integrating these technologies with antimicrobial stewardship programs (ASPs), reporting improved time to optimal therapy, higher de-escalation rates, and better clinical outcomes [7].

Despite growing interest, the effectiveness of RDTs in guiding antibiotic decision-making remains heterogeneous across settings. Differences in study design, patient populations, timing of test implementation, hospital resources, and integration with ASPs contribute to these variable outcomes [8]. Hence, there is a need for a systematic evaluation of randomized controlled trials (RCTs) to establish the role of RDTs in optimizing antimicrobial therapy in sepsis and bloodstream infections.

The objective of this systematic review is to critically evaluate and synthesize evidence from RCTs assessing the effectiveness of rapid diagnostic testing in guiding antibiotic therapy among patients with bloodstream infections or sepsis. This review aims to determine the clinical benefits, limitations, and implications of rapid diagnostics in improving antimicrobial stewardship and patient outcomes.

## Review

Materials and methods

Search Strategy and Information Sources

This systematic review was conducted in accordance with the Preferred Reporting Items for Systematic Reviews and Meta-Analyses (PRISMA) guidelines [9] to ensure methodological rigor and transparency. A comprehensive literature search was performed using major databases, including PubMed, Scopus, Embase, and the Cochrane Central Register of Controlled Trials, covering studies published up to July 2024. The search strategy incorporated keywords and Boolean combinations related to “rapid diagnostic tests,” “bloodstream infections,” “randomized controlled trials,” “antimicrobial stewardship,” and “clinical outcomes.” Additional records were identified by screening reference lists of relevant reviews and included articles to ensure a thorough capture of the available evidence. The search and selection process was structured according to the PICO framework [10]: the Population included hospitalized patients with suspected or confirmed bloodstream infections; the Intervention comprised rapid diagnostic tests such as multiplex PCR, MALDI-TOF MS, or rapid phenotypic antimicrobial susceptibility testing; the Comparator was conventional microbiological diagnostics; and the Outcomes assessed included time to effective therapy, time to organism identification, antibiotic optimization, mortality, and length of hospital stay (LOS).

Eligibility Criteria

Studies were selected based on predefined inclusion and exclusion criteria. Only RCTs and post-hoc analyses of RCTs that evaluated the impact of RDTs on the management of BSIs were considered eligible. Studies were required to compare any form of RDT, such as multiplex PCR, MALDI-TOF MS, or rapid phenotypic AST, with conventional diagnostic workflows, and report at least one clinical outcome relevant to antimicrobial therapy, such as time to antibiotic optimization, escalation or de-escalation, or overall treatment effectiveness. Observational studies, reviews, conference abstracts, and studies not published in English were excluded.

Study Selection and Data Extraction

Two independent reviewers screened titles and abstracts for initial eligibility, followed by full-text review for final inclusion. Disagreements were resolved through discussion or by consultation with a third reviewer. Data were extracted using a standardized form developed a priori. Extracted information included study author and year, design, population characteristics, sample size, type of RDT used, comparator intervention, and reported clinical outcomes. Specific attention was given to outcomes such as time to effective antimicrobial therapy, time to organism identification, antibiotic optimization rates, mortality, and LOS.

Risk of Bias (RoB) Assessment

The methodological quality of each included RCT was assessed independently by two reviewers using the Cochrane Risk of Bias (RoB 2.0) tool [11]. This tool evaluates several domains, including randomization process, deviations from intended interventions, missing outcome data, measurement of the outcome, and selection of the reported result. Each domain was rated as low, some concerns, or high RoB, and a summary judgment was assigned accordingly. Post-hoc analyses were also assessed using the same criteria with acknowledgment of their secondary nature.

Data Synthesis and Analytical Approach

Given the heterogeneity in outcome measures, study designs, and types of RDTs employed, a meta-analysis was not conducted. Instead, a narrative synthesis was undertaken to summarize findings across the selected studies. This synthesis aimed to highlight patterns, similarities, and differences in outcomes and methodological quality while considering the presence or absence of ASPs as a co-intervention. Where appropriate, comparisons were drawn across types of RDTs and patient populations to generate meaningful conclusions relevant to both high-income and low-resource settings.

Results

Study Selection Process

**Figure 1:**
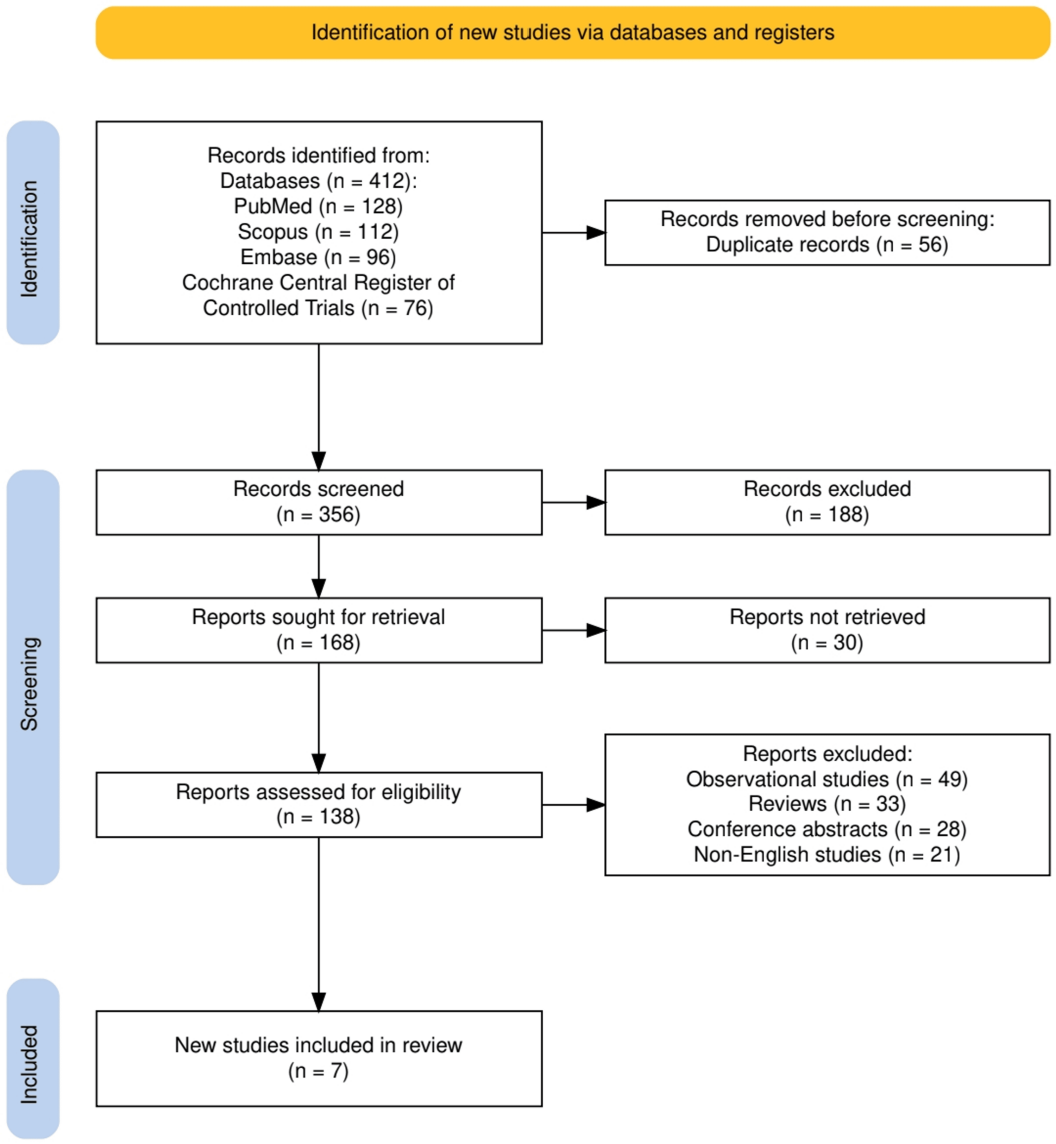
The PRISMA flowchart represents the study selection process PRISMA: Preferred Reporting Items for Systematic reviews and Meta-Analyses [9].

The study selection process is illustrated in Figure 1, which provides a PRISMA flow diagram outlining each phase of article identification, screening, eligibility assessment, and final inclusion.

A total of 412 records were identified through database searches: PubMed (n=128), Scopus (n=112), Embase (n=96), and the Cochrane Central Register of Controlled Trials (n=76). After removing 56 duplicate entries, 356 records remained for screening. Following title and abstract screening, 188 studies were excluded due to irrelevance to the study’s scope. Of the 168 full-text reports sought for retrieval, 30 could not be accessed, leaving 138 full-text articles assessed for eligibility. Ultimately, 131 articles were excluded due to not meeting inclusion criteria, comprising observational studies (n=49), reviews (n=33), conference abstracts (n=28), and non-English publications (n=21). A total of seven RCTs met all inclusion criteria and were included in the final synthesis of this systematic review.

Characteristics of the Selected Studies

Table 1 outlines the main characteristics of the included studies, all of which were RCTs or post-hoc analyses involving hospitalized patients with BSIs.

**Table 1:** Summary of the included studies in the review RCT: Randomized Controlled Trial; BCBs: Blood Culture Bottles; rmPCR: Rapid Multiplex Polymerase Chain Reaction; ASP: Antimicrobial Stewardship Program; ID: Identification; AST: Antimicrobial Susceptibility Testing; MALDI-TOF MS: Matrix-Assisted Laser Desorption Ionization–Time-of-Flight Mass Spectrometry; mPCR: Multiplex Polymerase Chain Reaction; ePlex®: A brand name for a multiplex PCR blood culture panel; SES: Severe Sepsis; FN: Febrile Neutropenia; SIE: Suspected Infective Endocarditis; RDT: Rapid Diagnostic Test.

Study (Author, Year)	Study design	Population	Sample size (N)	Type of RDT used	Comparator	Outcome(s)
Banerjee et al., 2015 [12]	RCT	Patients with positive BCBs	617	rmPCR with and without ASP	Standard blood culture processing	Time to antimicrobial de-escalation/escalation; Duration of broad-spectrum and narrow-spectrum antibiotic use
Banerjee et al., 2021 [13]	RCT	Patients with gram-negative bacilli bloodstream infections	448	Accelerate Pheno System (rapid organism ID and phenotypic AST) with ASP	Standard-of-care blood culture and AST with ASP	Time to first antibiotic modification within 72 hours (overall and gram-negative antibiotics); time to antibiotic escalation
MacGowan et al., 2020 [14]	RCT	Hospitalized adults with positive blood cultures	5550 (2740 rapid, 2810 conventional in final analysis)	MALDI-TOF MS performed directly on positive blood cultures	Conventional microbial identification methods	28-day mortality; time to microbial ID; time to effective antimicrobial therapy
Caspar et al., 2024 [15]	RCT	Hospitalized patients with bloodstream infections	212 (105 mPCR, 107 control)	ePlex® multiplex PCR blood culture identification panel	Conventional blood culture and reporting	Percentage of patients with optimized antimicrobial treatment within 12 hours of Gram stain result
Kim et al., 2022 [16]	Post-hoc RCT analysis	Hematological patients with bloodstream infections at high risk of poor outcomes	116	Rapid phenotypic AST with antimicrobial stewardship	Conventional AST with ASP	Proportion of patients receiving optimal targeted therapy at 72 hours post blood culture; time to optimal targeted therapy
Cambau et al., 2017 [17]	Cluster-randomized crossover trial	Patients with severe sepsis, febrile neutropenia, or suspected infective endocarditis	1416 (907 SES, 440 FN, 69 SIE)	LightCycler® SeptiFast molecular pathogen detection	Standard diagnostic workup	Microbiological diagnosis rate; turnaround time; cost-effectiveness
Nadjm et al., 2019 [18]	RCT	Patients with invasive bacterial or fungal infections (primarily bloodstream infections)	628 (326 intervention, 302 control)	MALDI-TOF MS for pathogen identification	Conventional microbiological identification methods	Proportion of patients receiving optimal antimicrobial therapy within 24 hours of positive culture

Sample sizes ranged from 116 to 5,550. The studies evaluated various RDTs, multiplex PCR, MALDI-TOF MS, and rapid phenotypic AST, against conventional diagnostics. Outcomes assessed included time to effective therapy, antimicrobial optimization, mortality, and organism identification. This table provides a concise overview of the study designs, interventions, and key clinical endpoints relevant to the review.

Quality Assessment

Table 2 presents the quality assessment of the included studies using the Cochrane RoB 2.0 tool.

**Table 2:** The risk of bias assessment of the included studies RoB: Risk of Bias; RCT: Randomized Controlled Trial; ASP: Antimicrobial Stewardship Program.

Study (Author, Year)	RoB tool applied	Randomization process	Deviations from intended interventions	Missing outcome data	Measurement of outcome	Selection of reported result	Overall risk of bias
Banerjee et al., 2015 [12]	RoB 2.0 (Parallel RCT)	Low risk	Low risk	Low risk	Low risk	Low risk	Low
Banerjee et al., 2021 [13]	RoB 2.0 (Parallel RCT)	Low risk	Low risk	Low risk	Low risk	Low risk	Low
MacGowan et al., 2020 [14]	RoB 2.0 (Multicenter Parallel RCT)	Low risk	Some concerns (intervention delivery not blinded)	Low risk	Low risk	Low risk	Some concerns
Caspar et al., 2024 [15]	RoB 2.0 (Single-center RCT)	Low risk	Low risk	Low risk	Low risk	Some concerns (early termination)	Some concerns
Kim et al., 2022 [16]	RoB 2.0 (Post-hoc RCT Analysis)	Some concerns (secondary analysis)	Low risk	Low risk	Low risk	Some concerns	Some concerns
Cambau et al., 2017 [17]	RoB 2.0 (Cluster-RCT, crossover)	Some concerns (cluster design complexities)	Some Concerns (open-label design)	Low risk	Low risk	Low risk	Some concerns
Nadjm et al., 2019 [18]	RoB 2.0 (Parallel RCT)	Low risk	Some Concerns (no ASP integration)	Low risk	Low risk	Low risk	Some concerns

The majority of studies were rated as low RoB across key domains such as the randomization process, outcome measurement, and completeness of data. However, some studies raised concerns due to factors like open-label designs, lack of blinding, secondary analyses, or the complexities of cluster-randomized designs. These methodological issues, while not significantly compromising overall quality, suggest that interpretations of certain findings should be made with careful consideration of potential biases.

Discussion

Our systematic review demonstrated that RDTs, particularly when coupled with ASPs, consistently reduced the time to pathogen identification and targeted antimicrobial therapy. Tools such as MALDI-TOF MS, Accelerate Pheno, ePlex, and rapid multiplex PCR showed marked improvements in accelerating diagnosis compared to conventional methods [19]. However, improvements in broader clinical outcomes such as mortality or LOS remained inconsistent across trials, with several studies unable to establish statistically significant reductions.

Among the evaluated tools, the Accelerate Pheno system (Banerjee et al., [13]) and MALDI-TOF MS (MacGowan et al., [14]; Nadjm et al., [18]) notably shortened the time to antimicrobial modification and organism identification, respectively. The integration of ASPs was crucial in translating these diagnostic gains into clinical action. For instance, Banerjee et al. [12] showed that the addition of ASPs to rapid PCR testing significantly improved the timing of antimicrobial escalation and de-escalation decisions. Kim et al. [16] further highlighted that high-risk hematologic patients achieved significantly greater rates of optimal targeted therapy within 72 hours when ASPs guided the use of rapid phenotypic AST. Despite these advantages, the ultimate impact on mortality varied: MacGowan et al. [14], using MALDI-TOF in a large cohort, found no significant mortality benefit, suggesting that faster diagnosis alone may be insufficient without comprehensive clinical integration. These findings underscored that while RDTs enhance diagnostic efficiency, their greatest value is realized when embedded in robust stewardship frameworks tailored to specific patient populations and care settings.

Previous systematic reviews and meta-analyses have consistently highlighted the benefits of RDT in reducing the time to appropriate antimicrobial therapy and improving clinical outcomes in BSIs [20]. For example, a meta-analysis by Timbrook et al. emphasized that RDTs, particularly when integrated with ASPs, led to reduced mortality and faster de-escalation of broad-spectrum antibiotics [21]. Our findings aligned with these conclusions, and built upon them by including only RCTs, thus providing more robust evidence. Additionally, while previous literature had grouped various RDT modalities together, our review disaggregated the tools and examined the impact of individual technologies, offering more granular insights into their clinical utility, implementation challenges, and context-dependent effectiveness.

A novel insight emerging from this review was the consistent observation that RDTs alone did not translate into improved patient outcomes unless their results were swiftly interpreted and acted upon, highlighting a diagnostic-therapeutic gap. This reinforced the concept that rapid identification was necessary but insufficient without coordinated clinical response, usually through ASP involvement [20]. Furthermore, the review shed light on the operational and infrastructural challenges that hindered the implementation of RDTs in low- and middle-income countries, such as cost, lack of trained personnel, and delayed turnaround times despite technical capabilities. Rather than focusing solely on mortality, which is often influenced by confounding variables, we advocated for more sensitive surrogate outcomes like time to de-escalation, antibiotic-free days, and LOS to more accurately assess the clinical impact of RDTs. This review also underscored the need for structured diagnostic stewardship, an emerging concept that encompasses appropriate test utilization, clinician education, and integration into therapeutic decision-making pathways.

This review possessed several strengths that enhanced its methodological rigor and relevance. By limiting inclusion to RCTs, we prioritized high-quality evidence and minimized biases inherent in observational studies. The use of a standardized and validated RoB assessment tool further strengthened the credibility of our analysis by ensuring transparent appraisal of methodological quality across included studies. Additionally, our review adhered strictly to PRISMA guidelines, providing a reproducible and well-documented framework for literature identification, screening, extraction, and synthesis. These methodological safeguards collectively ensured that our findings offered a dependable foundation for clinical decision-making and future research design.

Nonetheless, our review is subject to certain limitations that warrant acknowledgment. The heterogeneity of outcomes and interventions across trials posed a challenge in synthesizing results and precluded the conduct of a meaningful meta-analysis. Moreover, while the trials included were of high methodological quality, the majority originated from high-income healthcare systems, limiting the generalizability of findings to low- and middle-income countries, where diagnostic infrastructures and ASPs may be less robust. Additionally, although we employed comprehensive search strategies, the possibility of publication bias remains, particularly for studies with negative or neutral results. Despite these limitations, our findings offer valuable insights that should be interpreted within the context of these constraints.

The findings of this review have several important implications for clinical practice, health policy, and laboratory operations. Clinicians are encouraged to utilize RDTs not in isolation, but in conjunction with active ASPs to ensure that rapid results are translated into timely therapeutic decisions [22,23]. Policymakers should consider integrating RDTs into national antimicrobial stewardship frameworks and infection management guidelines, particularly in hospitals where sepsis burden is high. From a laboratory perspective, institutions should prioritize the adoption of cost-effective and operationally feasible RDT platforms based on local epidemiology, turnaround requirements, and available human resources. The value of these technologies lies not just in their speed, but in the systems surrounding their effective use [24].

Future research should aim to address existing knowledge gaps by conducting high-quality RCTs in resource-limited settings where the burden of sepsis is often the greatest and healthcare systems may struggle with implementing advanced diagnostics. Additionally, long-term cost-effectiveness analyses are needed to evaluate not only the clinical outcomes but also the economic sustainability of incorporating RDTs into routine care [25]. Research into the integration of clinical decision support systems that interface with diagnostic platforms could further enhance the utility of RDTs by promoting standardized, evidence-based responses to test results. These directions will help guide the evolution of diagnostic technologies from stand-alone tools to fully embedded components of patient-centered care.

## Conclusions

This systematic review underscored the pivotal role of RDTs in enhancing antimicrobial stewardship and optimizing clinical outcomes, particularly when integrated with structured implementation strategies. While RDTs offered the potential to significantly reduce time to appropriate therapy, their standalone use without real-time clinical intervention yielded limited benefit, a finding consistently emphasized across the included RCTs. Our review not only consolidated the existing high-quality evidence but also highlighted the need for diagnostic stewardship, context-specific adaptation, and improved surrogate outcomes beyond mortality. The variability in effectiveness across healthcare settings further illustrated the necessity for tailored approaches, especially in low-resource environments where barriers to implementation remain substantial. Ultimately, our findings advocate for a paradigm shift from merely deploying diagnostic tools to embedding them within a responsive, multidisciplinary care framework, ensuring that technological advances translate into meaningful improvements in patient care and public health outcomes.
